# Safety and Immunogenicity of Recombinant Adeno-Associated Virus-Vectored African Swine Fever Virus Antigens

**DOI:** 10.1155/vmi/9191117

**Published:** 2025-03-02

**Authors:** Antonina Galeeva, Marina Efimova, Gennadiy Frolov, Nail Khammadov, Almaz Hisamutdinov, Lenar Garipov, Danil Mingaleev, Rustam Ravilov

**Affiliations:** ^1^Interdepartmental Laboratory of Immunology and Biotechnology, Kazan State Academy of Veterinary Medicine Named After N.E. Bauman, Kazan, Russia; ^2^Laboratory of Viral Antropozoonoses, Federal Center for Toxicological, Radiation and Biological Safety, Kazan, Russia; ^3^Department of Microbiology, Kazan (Volga Region) Federal University, Kazan, Russia; ^4^Department of Epizootology and Parasitology, Kazan State Academy of Veterinary Medicine Named After N.E. Bauman, Kazan, Russia; ^5^Laboratory of Molecular Genetics, Federal Center for Toxicological, Radiation and Biological Safety, Kazan, Russia; ^6^Ministry of Agriculture and Food of Republic of Tatarstan, Kazan, Russia; ^7^Administrative Department, Federal Center for Toxicological, Radiation and Biological Safety, Kazan, Russia

## Abstract

Despite the significant global economic damage caused by African swine fever (ASF) and ongoing developments in the field of specific prevention tools development, safe and effective vaccines are still missing. A critical factor hindering the development of ASF vaccines is the lack of sufficient data on the pathogenesis of the virus, as well as a deep understanding of the virus' evasion strategies from the innate immune system. Of particular interest in the design of candidate vaccines are viral vectors, especially-adeno-associated virus (AAV), which is widely used in gene therapy and is capable of long-term transgene expression in vivo. This study assessed the safety and immunogenicity of recombinant AAV serotype 2 (rAAV2), into the genome of which the ASF virus *B646L* (p72)*, E183L* (p54)*, CP530R* (pp60), and *CP204L* (p30) immunodominant genes are integrated. The study design included immunization of pigs with monocistronic and bicistronic constructs based on rAAV2 in different regimens, assessment of the safety and tolerability of a laboratory sample of the vaccine, the biochemical and hematological status of the animals, as well as indicators of humoral and cellular immunity. It was found that rAAV2s in immunizing doses no more than 10 × 10^11^ viral particles have satisfactory tolerability, promote the formation of virus-specific antibodies that remain at a high level at least until the 180 days of the experiment. It has been proven that the use of bicistronic constructs makes it possible to achieve a similar immune response as when introducing a cocktail of monocistronic constructs, which allows to reduce the vector load on the animal's body. Thus, rAAV2 is a promising platform for the construction of a candidate vaccine against ASF, as it is biologically safe and activates the humoral and cellular immune response, which is extremely important for the formation of a protective immunity.

## 1. Introduction

African swine fever (ASF) is a viral hemorrhagic disease with exceptionally high mortality in representatives of the *Suidae* family (mainly domestic pigs and wild boars). Despite its limited host range and lack of zoonotic potential, the socioeconomic impact of this disease is very high [1]. ASF is a disease under the responsibility of the World Organization for Animal Health (WOAH), as it has serious economic consequences associated with production losses, trade restrictions and eradication programs [2]. Since 2020, ASF has been reported in five regions of the world, covering 45 countries, causing the death of approximately 2 million pigs [3]. The ASF outbreak has led to a sharp decline in global pig production capacity; according to different researchers, it may take three to 5 years to restore pigs number [4].

Despite the fact that ASF vaccine development research has been ongoing since the 1920s [5], the majority of currently available experimental vaccines, including subunit vaccines, DNA vaccines and virally vectored vaccines, did not provide a sufficient protective effect [6]. Some live attenuated vaccines have shown enhanced immune protection in comparison with inactivated and subunit vaccines, however, an efficacious, safe ASFV vaccine does currently not exist [7]. Development and innovation in prophylactic vaccines involve further progress in identification of viral antigens, improving delivery strategies, and increasing the knowledge on protective immune mechanisms [8].

Viral vectors are of particular interest in the design of candidate vaccines. In such vaccines, the genome of the viral particle contains one or more immunodominant genes of the target virus, which can induce a protective immune response [4, 9]. In addition, virus vector vaccines allow the differentiation of infected and vaccinated animals (DIVA serological approach) by using the immunogen encoded by the viral vector as a vaccine marker [6]. There is information about studies of the functionality of various viral tools for gene delivery, in particular, the potential of baculoviruses [10], adenoviruses [11, 12], lentiviruses [13], Newcastle disease virus [14] and others [15] has been studied. In our opinion, a promising tool for gene delivery is the adeno-associated virus (AAV), which has successfully proven itself as a gene therapy agent in humane medicine [16]. AAVs belonging to the *Dependoparvovirus* genus of the *Parvoviridae* family contain single-stranded DNA with inverted terminal repeat (ITR) and a protein capsid [17]. The main advantages of AAV include the ability to transduce dividing and nondividing cells, low immunogenicity and long-term expression of the transgene in vivo [18]. We previously reported the ability of genetic constructs based on AAV serotype 2 and the ASFV *B646L* (p72)*, E183L* (p54)*, CP204L* (p30)*, CP530R* (pp60) genes to efficient in vitro transduction of porcine cells [19].

The aim of this study was to assess the safety and immunogenicity of AAV serotype 2 carrying ASFV genes.

## 2. Materials and Methods

### 2.1. Study Period and Location

The study was carried out from June to December, 2023, in the Interdepartmental Laboratory of Immunology and Biotechnology (Kazan state academy of veterinary medicine, Kazan, Russia).

### 2.2. Genetic Constructs

The sequences of the *B646L, CP204L, E183L*, and *CP530R* genes, encoding the target proteins p72, p30, p54, pp62, respectively, were optimized in silico (the most frequently occurring codons of the recipient organism were used as the optimal). Genes containing the transgene and its regulatory elements flanked by ITRs were synthesized outsourced (Evrogen, Russia), and were cloned into the pAAV-MCS plasmid (Stratagene, USA). In addition to monoconstructs, we additionally created bicistronic constructs that combine the target genes by pairs, since the p54 and p30 proteins are involved in the process of internalization of the ASF virus into the cell, and p72 and pp62 are the dominant structural viral proteins and, as a consequence, targets for serological diagnostics. The target fragments in the bicistronic constructs were separated by a P2A linker; the general structure of the insertions is represented as: CMV-gene1-P2A-gene2-stop-polyA. Bicistronic inserts were cloned into the vector using the *Bam*HI and *Eco*RI restriction sites. The correctness of the cloning steps was confirmed by sequencing. Target mono- and bicistronic constructs, as well as envelope (pAAVRepCap2) and packaging (pHelper) plasmids (Stratagene, USA), necessary for the assembly of the viral capsid, were produced in *E. Coli* DH5*α* cells (Addgene, USA) transformed by heat shock, with ampicillin selection. Plasmid DNA was purified using a Midiprep system (Evrogen, Russia).

### 2.3. Assembly of Recombinant AAV2

To assemble recombinant AAV2 (rAAV2), we used the AAV293 cell line (ATCC CRL-1573, USA), a derivative of the human embryonic kidney cell line HEK293 that stably expresses the adenovirus *E1* gene. AAV293 cells were cultured in culture dishes (10 cm^2^) in DMEM medium (Paneco, Russia) supplemented with 10% bovine fetal serum (HyClone, Australia), 200 μM L-glutamine (Sigma-Aldrich, USA) and 100 IU/mL penicillin-streptomycin (Gibco, USA) at 37°C in 5% CO_2_ atmosphere to 70% monolayer density. Calcium phosphate transfection was carried out with three plasmids (with the gene of interest, envelope and packaging) at the rate of 10 mcg each per dish. After 6 h of incubation, the complete medium was replaced with a supporting one and incubated for up to 72 h. Additionally, rAAV2 was assembled, containing the far-red protein TurboFP635 gene for fluorescent control of cells transduction. After the incubation period, the medium was collected, the cells were removed mechanically and subjected to three-time cryolysis at −80°C, followed by reprecipitation. The rAAV2 titer was determined in the obtained samples: the samples were treated with Benzonase Nuclease (Sigma Aldrich, USA) at a rate of 50 U/mL and incubated at 37°С for 30 min to get rid of unencapsidated DNA. Then the samples were incubated at 95°С for 10 min to inactivate benzonase and destruct of viral capsids, after which quantitative RT-PCR was performed using primers and a probe for ITR, as well as specific primers flanking the target gene loci [18]; the reference was a plasmid containing the ITR locus in dilutions of 10^2^–10^8^. Additionally, the presence of rAAV2 in the samples was monitored by protein electrophoresis in a 12.5% polyacrylamide gel, and the major capsid protein VP3 (62 kDa) was visualized.

### 2.4. Purification of rAAV2

To concentrate the virus-containing material, a precipitation solution (2.5 M NaCl, 40% PEG-8000) was added to the cryolysate of transfected cells in a ratio of 1:4 and stored overnight, after which it was centrifuged at 3000 g for 30 min at 4°C. A 25% sodium deoxycholate solution was added to the mixture in a ratio of 1:50, mixed and incubated at 37°C for 30 min, and centrifuged at 4000 g for 30 min. Gradient purification was applied to the resulting supernatant. Iodixanol solutions at concentrations of 60%, 40%, 25%, 15% in a volume of 10 cm^3^ each were sequentially layered into 50 cm^3^ volume ultracentrifuge tubes, and 10 cm^3^ of virus-containing supernatant was layered on top. The tubes were centrifuged at 200,000 g at 10°С for 2 h on an Optima X-100 centrifuge (Beckman, USA) using an SW 41 Ti swing-bucket rotor. After centrifugation, the walls of the tubes were punctured and a fraction of 40%–60% was aspirated and diluted with Ringer's solution in a ratio of 1:2. The rAAV2 titer was further determined in purified samples.

### 2.5. Validation of Protein Expression by the rAAV2 Constructs

The functionality of the developed constructs was evaluated by the specific expression of the transgene (determination of the relative levels of messenger RNA [mRNA]) and by the presence of expression products in cell lysates (mature proteins p72, p30, p54, and pp62). Transduction of SPEV cell line was performed as described previously [19]. Total RNA was isolated from the cells using TRI reagent, and the upper colorless aqueous phase was removed after precipitation. After measuring the RNA concentration, a matrix was used for complementary DNA synthesis. After reverse transcription, real-time PCR was performed using the above primers and the 10-fold serially diluted plasmids with the corresponding transgenes as references.

### 2.6. Animal Experiments and Ethics Statement

Both female and male pigs of a large white breed 3 months old weighing 35–40 kg from the epizootic-free pig farm (Republic of Tatarstan, Russia) were used. Experiments involving animals were performed in accordance with the National Institutes of Health's Guide for the Care and Use of Laboratory Animals, and ARRIVE Guidelines [20, 21], and were approved by the institutional local ethics committee (ethics approval no. 3, 04/2023). All efforts were made to minimize suffering. The scheme of the experiment is provided in Table 1.

Pigs of all experimental groups were immunized intramuscularly into a trapezoid muscle of the neck with rAAV2-containing constructs in a final volume 2.0 mL of phosphate-buffered saline (PBS) as described above. The body temperature of the pigs was measured rectally daily. During the experiment, blood samples were collected from the *vena auricularis posterior* on days 0, 15, 30, 45, 60, 90, and 180. Blood was taken into test tubes with a coagulant for receiving serums and test tubes with anticoagulant for PBMCs analysis.

### 2.7. rAAV2 Safety Assessment

The reactogenicity of rAAV2 was assessed by the presence of local and systemic postvaccination reactions, its severity and duration based on active monitoring of pigs for 14 days. The severity of local reactions was assessed by the intensity of hyperemia and swelling at the injection site of the laboratory vaccine sample. Systemic reactions were assessed by the degree of hyperthermia and the severity of manifestations of intoxication. The safety of rAAV2 was assessed based on the hematological and biochemical status of pigs during vaccination. To assess the effect of rAAV2 on the functionality of internal organs, biochemical profiles of the liver, heart and kidney were studied.

### 2.8. Detecting of Virus-Specific Antibodies

Serum samples were tested in triplicates using « ID Screen African Swine Fever Indirect » kit (Ingenasa, Spain). According to manufacturer`s instructions, the status of each tested serum was expressed using the coefficient of inhibition (x %).

To assess the dynamics of antibody formation for each of the expressed antigens, indirect ELISA was also performed separately with recombinant purified ASFV proteins (Creative Diagnostics, USA): p54 (DAGC011), p72 (DAG-WT203), p30 (DAGC010), and pp62 (DAGC586). 96-well ELISA microtiter plates coated with each recombinant protein at concentrations from 5 to 10 ng per well diluted in carbonate buffer were incubated overnight at 4°C. After incubation, plates were washed three times with PBST containing 0.05% (*v*/*v*) Tween 20, blocked in 5% nonfat dry milk in PBST for 1 h at 37°C, and then rinsed one more time. Serum samples, including ASFV positive and negative controls, were diluted 1:100 in PBST, added to each well and incubated for 1 h at 37°C. After washing, 100 μL of 1:30,000 diluted HRP-conjugated anti-pig antibody (Sigma, USA) was added to each well, then incubated for 45 min at 37°C. The plates were washed 3 times, and 100 μL of tetramethylbenzidine (TMB) substrate (Thermo Scientific, USA) was added and incubated for 20 min at room temperature. Finally, 100 μL of 0.3 M H_2_SO_4_ was added to each well to determine the optical density (OD_450_), and the condition with the highest ratio of positive and negative sera (*P*/*N* value) was selected as the optimal working condition. The OD_450_ values of each serum sample were measured and interpreted using the formula: *X* = ((Sample OD − Negative control OD)/(Positive control OD − Negative control OD)). The cutoff value was determined according to ROC curve analysis.

### 2.9. Statistical Analysis

All experiments were repeated three times with consistent results. Data in figures are expressed as mean ± standard deviation (SD). The statistical significance of differences between groups was determined by one-way analysis of variance and Student's *t*-tests (GraphPad Prism software, San Diego, CA, USA). Values at ⁣^∗^*p* < 0.05, ⁣^∗∗^*p* < 0.01, and ⁣^∗∗∗^*p* < 0.001 were considered to be statistically significant differences between groups.

## 3. Results

### 3.1. Validation of Functionality of rAAV2 Constructs

When assessing the in vitro functionality of rAAV2 constructs, it was found that both monocistronic and bicistronic constructs support a fairly high level of specific mRNAs expression. The highest relative level of specific mRNAs expression during transduction of SPEV cells averaged from 7.1 to 9.7 million copies of the target gene/1 μg of total RNA, accompanied by the presence of mature proteins and fusion forms in cell lysates. It was found that in the bicistronic constructs, each upstream cistron was expressed comparably to the same transgene in the monocistronic constructs, whereas the level of expression of the downstream cistron was slightly lower and varied from 2.7 to 4.3 million copies of the target gene/1 μg of total RNA (Table 2). However, the data obtained allow assess the functional activity of the lower cistrons positively. Given the confirmed expression for both types of constructs, the material was prepared for pigs immunization.

### 3.2. rAAV2 Safety Assessment

**Figure 1:**
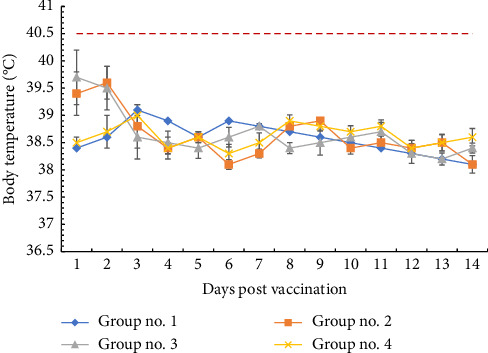
Daily body temperatures of animals after rAAV2 vaccination. Data are presented as group mean values (expressed as °C) for each experimental groups. The red dotted line marks the threshold for fever at 40.5°C. Error bars indicate standard deviation.

It was found that animals of all experimental groups remained healthy after both prime and boost vaccination. Body temperature indicators slightly increased by one to two dpv in animals of groups no. 2 and 3, however, did not overcome the febrile threshold (40.5°C) and were not statistically different from the indicators of animals in the control group until the end of the observation period (Figure 1).

When assessing the hematological status, a tendency toward neutrophilic leukocytosis was revealed in all vaccinated animals: thus, the number of segmented neutrophils in groups no. 1, 2, and 3 increased by 1.6 (*p* < 0.05), 1.51 (*p* < 0.05) and 1.57 (*p* < 0.05) times respectively. There was also a significant increase in the number of monocytes by 2.3 times (*p* < 0.001) relative to the control group. No statistically significant changes in erythrocyte indices were detected.

When assessing the biochemical status of vaccinated animals, no changes in the profiles of the liver, kidney and heart were also observed. However, it was found that vaccinated animals demonstrated significantly higher levels of C-reactive protein: this indicator was recorded in animals of groups no. 2 and 3, subjected to increased vector load (a cocktail of monocistronic rAAV2 at a dosage of 5.6 × 10^11^ v.p. in group no. 2 and bicistronic rAAV2 at a dosage of 2.8 × 10^11^ v.p. in group no. 3). The distribution of the studied indicators is presented in Table 3.

### 3.3. Dynamics of Virus-Specific Antibodies Formation

**Figure 2:**
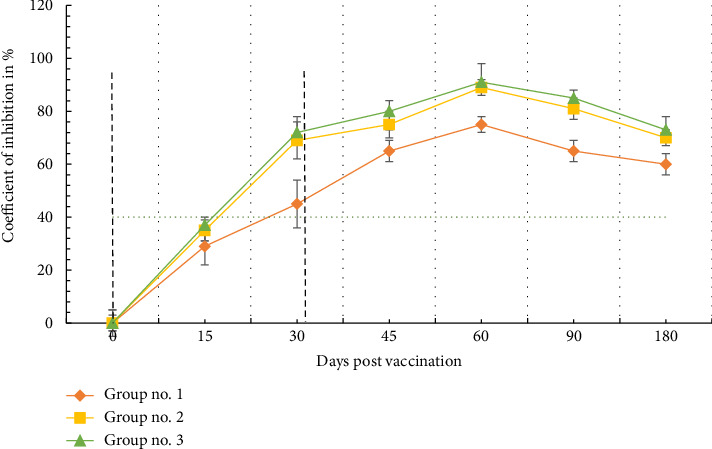
Evolution of virus-specific antibodies level in animals after rAAV2 vaccination. Data are presented as group values. The green dotted line marks inhibition threshold, black dotted lines-days of vaccination. Error bars indicate standard deviation.

Animals of all experimental groups responded to rAAV2 administration by producing virus-specific antibodies (Figure 2). 15 days dpv, animals from all three experimental groups had a doubtful serological status (inhibition coefficient from 30% to 40%), but by 30 dpv all animals became seropositive. An increase in antibody levels after boost vaccination was observed up to 60 dpv and slightly decreased by 180 dpv. The lowest endpoint antibody level was observed in group no. 1, the animals in which received a cocktail of monoconstructs at a total dosage of 2.8 × 10^11^ v.p.: the average inhibition coefficient in this group did not exceed 75%. In groups no. 2 and 3, the animals in which were subjected to increased vector load, this indicator varied between 89% and 91%; there were no significant differences in antibody levels between these groups.

**Figure 3:**
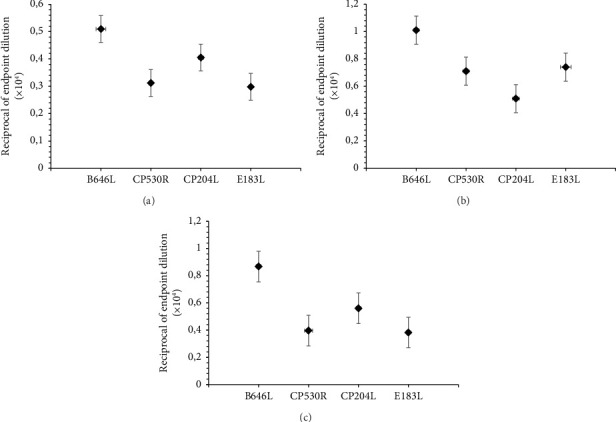
Evolution of virus-specific antibodies levels separately to each antigen in animals after rAAV2 vaccination: (a) group no. 1, (b) group no. 2, (c) group no. 3. Data are presented as group values.

Additionally, we studied the dynamics of antibody formation for each of the expressed proteins separately (Figure 3). The results of ELISA with individual recombinant antigens revealed different levels of specific antibodies depending on the levels of expression of these antigens by various constructs.

## 4. Discussion

It is known that initial research into the use of AAV for vaccine development was carried out using AAV serotype 2, which, despite the low transduction efficiency relative to other serotypes, induced the most powerful immune responses against the human immunodeficiency virus (HIV), the severe acquired respiratory syndrome coronavirus (SARS-CoV), and the human papillomavirus (HPV) [22, 23]. The choice of the introduction route of the AAV2-based genetic constructs was also dictated by the proven ability of the most effectively transgene expression a single dose of intramuscular injection [24, 25]. AAV2s are among the safest viral vectors in use, however the levels of humoral and cellular immune responses elicited by different doses in different animal species remain controversial [26]. Taking this into account, our first task was to assess the preliminary safety profile of a candidate vaccine based on AAV2 carrying the ASFV *B646L*, *E183L*, *CP204L,* and *CP530R* genes in different combinations. In the present study, we chose doses of 2.8 × 10^11^ v.p. and 5.6 × 10^11^ v.p. for a cocktail of monocistronic constructs and a dose of 2.8 × 10^11^ v.p. for a cocktail of bicistronic constructs containing pairwise combined genes *B646L-E183L* and *CP530R-CP204L*. It was found that prime and boost vaccination were satisfactorily tolerated by animals of all experimental groups. A postvaccination reaction at one to two dpv, including a moderate increase in temperature ((1.0 ± 0.2) °C), was observed in animals that received an increased vector load (groups no. 2 and 3). It should be noted that in preliminary studies we tested higher doses of rAAV2 (up to 11.2 × 10^11^ v.p. for a cocktail of monocistronic constructs), but in this case postvaccination reactions were more pronounced and included an increase in temperature to subfebrile at 12–14 dpv, as well as petechial skin rash that disappeared independently within 1–2 days. Such delayed effects may indicate an allergic reaction caused by the process of expression of target antigens, and therefore increased doses were not used in the main experiment. Satisfactory tolerability of rAAV2 is also evidenced by the absence of changes in the biochemical profiles of the kidney, heart and liver; an increase in C-reactive protein level in animals of some experimental groups, in our opinion, may be caused by a moderate proinflammatory reaction to viral capsids, which is known to be a characteristic of intramuscular administration [27]. Administered rAAV2 has been shown to stimulate both humoral and cellular immune responses. Today, there is strong evidence of the role of both components of the adaptive response in protection against ASF virus [28], which explains modern approaches to the design of recombinant vaccines [4]. In the present study, it was found that the highest concentration of antibodies (inhibition coefficient-89%–91%) is achieved at approximately 60 dpv when vaccinated with a cocktail of monocistronic constructs at a dose of 5.6 × 10^11^ v.p. and bicistronic constructs-at a dose of 2.8 × 10^11^ v.p. Due to the absence of significant differences between these two groups, it can be argued that coexpression of two or more antigens allows for a reduction in vector load, which is especially important when immunization with a pool of antigens is necessary. However, the specific mechanisms of ASF virus neutralization by antibodies remain a controversial issue that requires clarification [29]. However, in prospective, the final characterization of viral vectors can only be made after assessing cellular immunity.

## 5. Conclusions

This study contains data on a preliminary assessment of the safety and immunogenicity of mono- and bicistronic constructs based on AAV2 and immunodominant ASFV genes—*B646L* (p72)*, E183L* (p54)*, CP204L* (p30), and *CP530R* (pp60). During the studies, it was found that the proposed designs are safe and easily tolerated by animals and cause the induction of humoral and immune response, however, a comprehensive characterization of the AAV2-based vaccine candidate can be compiled after lethal infection. The determinants of the balance between tolerability and immunogenicity of AAV2 have not been fully studied, so further development of the topic lies in the area of optimizing the mechanism for delivering target genes to the cells of the host organism, as well as searching for additional conservative antigens, which will be especially relevant when designing an economical and effective vaccine for the regions, endemic for ASF and characterized by high genetic diversity of circulating strains.

## Figures and Tables

**Table 1:** Experimental groups of animals for assessment of safety and immunogenicity of vaccination by rAAV2 vectored constructs.

Group no.	Prime vaccination (day 0)	Boost vaccination (day 30)
1 (*n* = 4)	rAAV2/*B646L*, rAAV2/*E183L*, rAAV2/*CP530R*, rAAV2/*CP204L* in equal parts, in total 2.8 × 10^11^ v.p	Similarly
2 (*n* = 4)	rAAV2/*B646L*, rAAV2/*E183L*, rAAV2/*CP530R*, rAAV2/*CP204L* in equal parts, in total 5.6 × 10^11^ v.p	Similarly
3 (*n* = 4)	rAAV2/*B646L-E183L*, raav2/*CP530R-CP204L* in equal parts, in total 2.8 × 10^11^ v.p	Similarly
4 (*n* = 4)	Empty vectored rAAV2, in total 5.6 × 10^11^ v.p	Similarly

**Table 2:** mRNA expression levels during validation of functionality of rAAV2 constructs.

Transgene	Copies of the target gene (million/mg of total RNA)	
	In monocistronic constructs	In bicistronic constructs
B646L	9.6 ± 0.7	9.2 ± 0.4 (as an upstream cistron)
CP530R	8.3 ± 0.9	4.3 ± 0.2 (as a downstream cistron)
CP204L	7.1 ± 0.5	6.7 ± 0.3 (as an upstream cistron)
E183L	7.4 ± 0.8	2.7 ± 0.2 (as a downstream cistron)

**Table 3:** Biochemical and hematological parameters of pig blood serum on the day 30 after rAAV2 introduction.

Parameter	Group no.			
	1	2	3	4
Erythrocytes, 10^12^/L	8.84 ± 0.42	8.97 ± 0.23	8.28 ± 0.32	9.44 ± 0.24
Hemoglobin, g/L	171.67 ± 5.12	161.67 ± 8.13	152.0 ± 4.64⁣^∗^	180.67 ± 0.17
Hematocrit, %	49.97 ± 1.10	48.6 ± 0.62	49.53 ± 3.09	54.32 ± 2.34
Mean corpuscular volume (MCV), fL	55.42 ± 1.85	55.84 ± 1.6	58.43 ± 1.62	54.42 ± 2.12
Mean concentration hemoglobin (MCH), pg	18.0 ± 0	18.33 ± 0.41	18.67 ± 0.41	18.41 ± 0.27
Mean corpuscular hemoglobin concentration (MCHC), g/L	330.0 ± 4.24	315.0 ± 11.68	326.0 ± 4.9	312.45 ± 6.71
RBC distribution, %	14.53 ± 0.11	14.77 ± 0.23	14.2 ± 0.21	14.18 ± 0.17
Leukocytes, ⁣^∗^10^9^/L	11.67 ± 1.44	11.86 ± 0.27	12.22 ± 1.04⁣^∗^	8.63 ± 0.34
Blasts, %	0	0	0	0
Myelocytes, %	0	0	0	0
Metamyelocytes, %	0	0	0	0
Band neutrophils, %	0	0	0	0
Segmented neutrophils, %	41.67 ± 2.27⁣^∗∗^	39.33 ± 2.16⁣^∗∗^	40.33 ± 1.47⁣^∗∗^	29.05 ± 3.65
Segmented neutrophils, 10^9^/L	4.68 ± 0,4⁣^∗^	4.22 ± 0.37⁣^∗^	4.9 ± 0.11⁣^∗∗^	2.79 ± 0.34
Eosinophils, %	0	11.33 ± 0.82	8.67 ± 4.32	8.14 ± 2.67
Eosinophils, 10^9^/L	0	1.26 ± 0.08	1.23 ± 0.12	1.74 ± 0.16
Basophils, %	0	0	0	0
Monocytes, %	3.0 ± 0.71	3.33 ± 0.82	7.67 ± 0.41⁣^∗∗∗^	3.14 ± 0.25
Monocytes, 10^9^/L	0.26 ± 0.08	0.38 ± 0.13	0.82 ± 0.08⁣^∗∗^	0.33 ± 0.05
Lymphocytes, %	52 ± 5.66	42.67 ± 5.35	62.0 ± 2.55	68.47 ± 3.21
Lymphocytes,10^9^/L	5.33 ± 0.42	5.67 ± 0.14	7.87 ± 0.44	5.98 ± 0.35
Plasmocytes, %	0	0	0	0
Thrombocytes, 10^9^/L	390.67 ± 27.07	416.33 ± 20.7	391.33 ± 14.45	452.45 ± 36.76
Mean platelet volume, fL	11.03 ± 0.67	11.23 ± 0.35	11.17 ± 0.5	11.64 ± 0.54
Reticulocytes, %	1.97 ± 0.4	1.9 ± 0.28	2.23 ± 0.25	1.83 ± 0.21
Normocytes, per 100 leukocytes	0	0	0	0
ESR, mm/h	0.97 ± 0.11	1.07 ± 0.15	1.07 ± 0.15	1.02 ± 0.19
C-reactive protein	Negative	Positive⁣^∗^	Positive⁣^∗^	Negative
Urea, mM/L	5.27 ± 0.25	5.5 ± 0.44	4.63 ± 0.29	5.63 ± 0.23
Creatinin, mcM/L	125 ± 5.34	116.4 ± 0.64	128.67 ± 2.95	151.61 ± 3.6
Total bilirubin, mcM/L	3.8 ± 0.28	3.23 ± 0.43	2.37 ± 0.6	2.39 ± 0.21
Direct bilirubin, mcM/L	1 ± 0.18	0.8 ± 0.14	0.8 ± 0.25	0.96 ± 0.29
Aspartat aminotransferase, U/L	31.33 ± 0.58	39.03 ± 0.7	35.66 ± 2.11	34.43 ± 0.51
Alanil aminotransferase, U/L	38.16 ± 0.41	34.13 ± 0.43	36.63 ± 3.9	42.5 ± 0.26
Alkaline phosphatase, U/L	122.33 ± 4.6	103.66 ± 2.94	139 ± 3.93	127.45 ± 4.31
Glucose, mM/L	4.95 ± 0.19	4.99 ± 0.21	4.72 ± 0.17	5.71 ± 0.25

*Note:* Variables of Significance (^∗^p ≤ 0.05, ^∗∗^p ≤ 0.01, ^∗∗∗^p ≤ 0.001).

## Data Availability

The data used and/or analyzed in this study are available from the corresponding author upon reasonable request.

## References

[1] Blome S., Franzke K., Beer M. (2020). African Swine Fever-A Review of Current Knowledge. *Virus Research*.

[2] Brown V. R., Bevins S. N. (2018). A Review of African Swine Fever and the Potential for Introduction Into the United States and the Possibility of Subsequent Establishment in Feral Swine and Native Ticks. *Frontiers in Veterinary Science*.

[3] Oie and Fao (2021). Global Control of African Swine Fever. A GF-TADs Initiative-2020 Annual Report.

[4] Zhang H., Zhao S., Zhang H., Qin Z., Shan H., Cai X. (2023). Vaccines for African Swine Fever: An Update. *Frontiers in Microbiology*.

[5] Netherton C. L., Goatley L. C., Reis A. L. (2019). Identification and Immunogenicity of African Swine Fever Virus Antigens. *Frontiers in Immunology*.

[6] Gaudreault N. N., Richt J. A. (2019). Subunit Vaccine Approaches for African Swine Fever Virus. *Vaccines*.

[7] Liu L., Wang X., Mao R. (2021). Research Progress on Live Attenuated Vaccine Against African Swine Fever Virus. *Microbial Pathogenesis*.

[8] Chathuranga K., Lee J.-S. (2023). African Swine Fever Virus (ASFV): Immunity and Vaccine Development. *Vaccines*.

[9] Zhou X., Lu H., Wu Z. (2022). Comparison of Mucosal Immune Responses to African Swine Fever Virus Antigens Intranasally Delivered With Two Different Viral Vectors. *Research in Veterinary Science*.

[10] Argilaguet J. M., Pérez-Martín E., López S. (2013). BacMam Immunization Partially Protects Pigs Against Sublethal Challenge With African Swine Fever Virus. *Antiviral Research*.

[11] Lokhandwala S., Waghela S. D., Bray J. (2017). Adenovirus-vectored Novel African Swine Fever Virus Antigens Elicit Robust Immune Responses in Swine. *PLoS One*.

[12] Zajac M. D., Trujillo J. D., Yao J. (2023). Immunization of Pigs With Replication-Incompetent Adenovirus-Vectored African Swine Fever Virus Multi-Antigens Induced Humoral Immune Responses But No Protection Following Contact Challenge. *Frontiers in Veterinary Science*.

[13] de Oliveira V. L., Almeida S. C. P., Soares H. R., Crespo A., Marshall-Clarke S., Parkhouse R. M. E. (2011). A Novel TLR3 Inhibitor Encoded by African Swine Fever Virus (ASFV). *Archives of Virology*.

[14] Chen X., Yang J., Ji Y. (2016). Recombinant Newcastle Disease Virus Expressing African Swine Fever Virus Protein 72 is Safe and Immunogenic in Mice. *Virologica Sinica*.

[15] Ravilov R. K., Rizvanov A. A., Mingaleev D. N. (2022). Viral Vector Vaccines Against ASF: Problems and Prospectives. *Frontiers in Veterinary Science*.

[16] Wang D., Tai P. W. L., Gao G. (2019). Adeno-Associated Virus Vector as a Platform for Gene Therapy Delivery. *Nature Reviews Drug Discovery*.

[17] Pillay S., Meyer N., Puschnik A. (2016). An Essential Receptor for Adeno-Associated Virus Infection. *Nature*.

[18] Deyle D. R., Russell D. W. (2009). Adeno-Associated Virus Vector Integration. *Current Opinion in Molecular Therapeutics*.

[19] Ravilov R., Galeeva A., Frolov G. (2023). Efficient Delivery of the Immunodominant Genes of African Swine Fever Virus by Adeno-Associated Virus Serotype 2. *Veterinary World*.

[20] Guide for the Care and Use of Laboratory Animals (2011). *National Research Council (US) Committee for the Update of the Guide for the Care and Use of Laboratory Animals*.

[21] Percie du Sert N., Ahluwalia A., Alam S. (2020). Reporting Animal Research: Explanation and Elaboration for the ARRIVE Guidelines 2.0. *PLoS Biology*.

[22] Nieto K., Salvetti A. (2014). AAV Vectors Vaccines Against Infectious Diseases. *Frontiers in Immunology*.

[23] Zabaleta N., Dai W., Bhatt U. (2021). An AAV-Based, Room-Temperature-Stable, Single-Dose COVID-19 Vaccine Provides Durable Immunogenicity and Protection in Non-Human Primates. *Cell Host & Microbe*.

[24] Liao G., Lau H., Liu Z. (2022). Single-Dose rAAV5-Based Vaccine Provides Long-Term Protective Immunity Against SARS-CoV-2 and Its Variants. *Virology Journal*.

[25] Tosolini A. P., Sleigh J. N. (2020). Intramuscular Delivery of Gene Therapy for Targeting the Nervous System. *Frontiers in Molecular Neuroscience*.

[26] Grimm D., Lee J. S., Wang L. (2008). In Vitro and in Vivo Gene Therapy Vector Evolution Via Multispecies Interbreeding and Retargeting of Adeno-Associated Viruses. *Journal of Virology*.

[27] Mingozzi F., High K. A. (2017). Overcoming the Host Immune Response to Adeno-Associated Virus Gene Delivery Vectors: The Race Between Clearance, Tolerance, Neutralization, and Escape. *Annual Review of Virology*.

[28] Goatley L. C., Nash R. H., Andrews C. (2022). Cellular and Humoral Immune Responses After Immunisation With Low Virulent African Swine Fever Virus in the Large White Inbred Babraham Line and Outbred Domestic Pigs. *Viruses*.

[29] Silva E. B., Krug P. W., Ramirez-Medina E. (2022). The Presence of Virus Neutralizing Antibodies is Highly Associated With Protection Against Virulent Challenge in Domestic Pigs Immunized With ASFV Live Attenuated Vaccine Candidates. *Pathogens*.

